# Bacterial Metabolism During Biofilm Growth Investigated by ^13^C Tracing

**DOI:** 10.3389/fmicb.2018.02657

**Published:** 2018-11-20

**Authors:** Ni Wan, Hao Wang, Chun Kiat Ng, Manisha Mukherjee, Dacheng Ren, Bin Cao, Yinjie J. Tang

**Affiliations:** ^1^Mechanical Engineering and Materials Science, Washington University, St. Louis, MO, United States; ^2^Department of Biomedical and Chemical Engineering, Syracuse Biomaterials Institute, Syracuse University, Syracuse, NY, United States; ^3^School of Civil and Environmental Engineering, Nanyang Technological University, Singapore, Singapore; ^4^Singapore Centre for Environmental Life Sciences Engineering, Nanyang Technological University, Singapore, Singapore; ^5^Department of Civil and Environmental Engineering, and Biology, Syracuse Biomaterials Institute, Syracuse University, Syracuse, NY, United States; ^6^Energy, Environmental and Chemical Engineering, Washington University, St. Louis, MO, United States

**Keywords:** c-di-GMP, dynamic labeling, Entner-Doudoroff pathway, pyruvate shunt, tubular biofilm reactors

## Abstract

This study investigated the metabolism of *Pseudomonas aeruginosa* PAO1 during its biofilm development via microscopy imaging, gene expression analysis, and ^13^C-labeling. First, dynamic labeling was employed to investigate glucose utilization rate in fresh biofilms (thickness 40∼60 micrometer). The labeling turnover time of glucose-6-P indicated biofilm metabolism was substantially slower than planktonic cells. Second, PAO1 was cultured in continuous tubular biofilm reactors or shake flasks. Then ^13^C-metabolic flux analysis of PAO1 was performed based on the isotopomer patterns of proteinogenic amino acids. The results showed that PAO1 biofilm cells during growth conserved the flux features as their planktonic mode. (1) Glucose could be degraded by two cyclic routes (the TCA cycle and the Entner-Doudoroff-Embden-Meyerhof-Parnas loop) that facilitated NAD(P)H supplies. (2) Anaplerotic pathways (including pyruvate shunt) increased flux plasticity. (3) Biofilm growth phenotype did not require significant intracellular flux rewiring (variations between biofilm and planktonic flux network, normalized by glucose uptake rate as 100%, were less than 20%). (4) Transcription analysis indicated that key catabolic genes in fresh biofilm cells had expression levels comparable to planktonic cells. Finally, PAO1, *Shewanella oneidensis* (as the comparing group), and their c-di-GMP transconjugants (with different biofilm formation capabilities) were ^13^C-labeled under biofilm reactors or planktonic conditions. Analysis of amino acid labeling variances from different cultures indicated *Shewanella* flux network was more flexibly changed than PAO1 during its biofilm formation.

## Introduction

Biofilm is a heterogeneous and dynamic system. Its development consists of steps of adhesion of planktonic microbes, colony formation and growth, and detachment/migration of dispersed cells to new surfaces. Moreover, cells at different locations inside a biofilm may have distinct metabolisms (e.g., different transcriptomic and proteomic profiles) due to intrinsic chemical gradients (Williamson et al., 2012). The physiological differences between biofilm and planktonic cells have attracted extensive studies (O’Toole et al., 2000; Bester et al., 2005). To quantify biofilm physiologies, diverse technologies including crystal violet assay, transcription/protein/metabolite analyses, and imaging (e.g., SEM, TEM, confocal microscopy) have been applied (Pantanella et al., 2013). Moreover, genetic mutations are used to reveal regulatory mechanisms of cell survival in various biofilm environments (Ding et al., 2014; Zhang et al., 2014). However, there is still little knowledge of metabolic fluxomes that describe *in vivo* enzyme activities inside biofilm cells for carbon/energy metabolism.

To decipher flux distributions in biofilm cells, the present study investigated the opportunistic pathogen *Pseudomonas aeruginosa* PAO1 for its metabolic functions under both planktonic and biofilm modes. Particularly, ^13^C-fingerprinting of proteinogenic amino acids was used to trace carbon fluxes for substrate utilization and biomass synthesis. In parallel, dynamic labeling via ^13^C-glucose pulses was used to reveal the speed of ^13^C percolating through central pathways in fresh biofilms as well as planktonic cells. This study also examined the c-di-GMP transconjugant of PAO1 via ^13^C-fingerprinting. The transconjugant overexpressed c-di-GMP and produced excess extracellular polymer substances (EPS) to enhance the biofilm formation (Chua et al., 2015). To broaden our perception of the degree of flux profile conservations between planktonic and biofilm cells, the same isotopic approaches were also used to investigate *Shewanella oneidensis* MR-1 (a metal reducing bacterium capable of proliferating in both aerobic and anaerobic conditions) (Tang et al., 2007). The outcomes improved our understanding of the mechanisms about how bacterial species reorganized their flux network during biofilm development.

## Materials and Methods

### Strains and Cultivations

**FIGURE 1:**
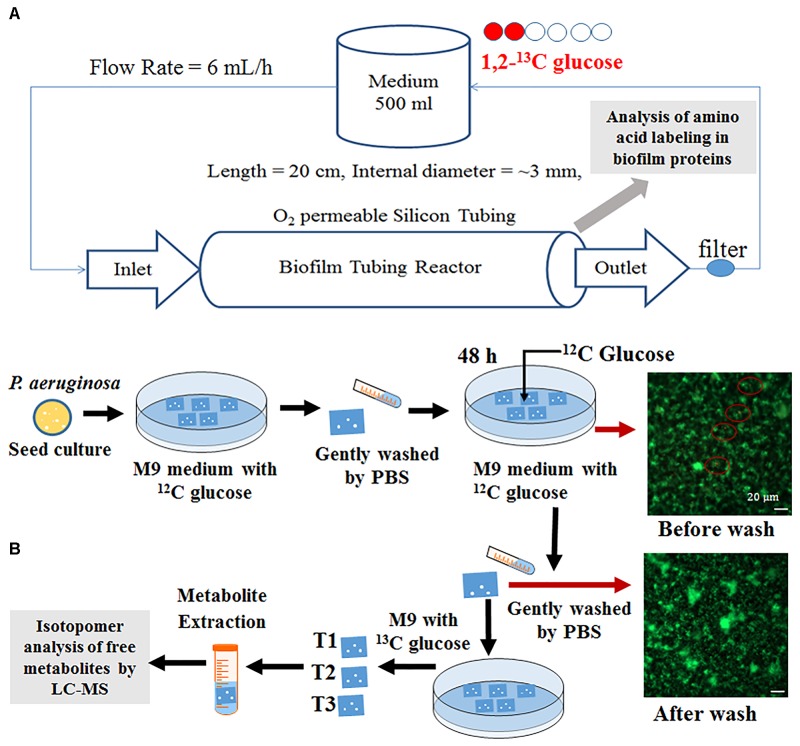
Experimental platform schematic illustration of **(A)** tubular biofilm reactor and **(B)** dynamic labeling in Petri dish system. Microscopic analysis showed majority cells on glass slides before labeling experiments were biofilm (in green) rather than planktonic cells (in orange, highlighted by red circles).

*Pseudomonas aeruginosa* PAO1 and its c-di-GMP transconjugants (i.e., a high c-di-GMP transconjugant with twice more biofilm formation and a low c-di-GMP transconjugant with reduced biofilm formation by ∼30%) were grown in an M9 medium using 20 mM glucose. *S. oneidensis* MR-1 and its high c-di-GMP transconjugant for enhanced biofilm formation, as additional tests, were grown in a modified MR-1 medium (Cao et al., 2011) using 20 mM sodium lactate. For planktonic cultures, bacteria (20 mL) were grown in flasks (150 mL) with inoculation volume ratio of 0.5% (at the room temperature, shaking at 200 rpm). To produce sufficient biofilm biomass for metabolic flux analysis (Figure 1), PAO1 or MR-1 was grown in tubular biofilm reactors (sets of 20 cm long O_2_-permeable silicon tubing with an inner diameter of 3 mm) at the room temperature (Ding et al., 2014), where the respective media were continuously pumped through the tubular reactor by Low-Speed Digital Peristaltic Pump system (Cole-Parmer, Singapore) (Sternberg and Tolker-Nielsen, 2006). Each tubular reactor was inoculated by injecting diluted planktonic culture using a syringe and resulted in initial OD_600_ of ∼0.01. After inoculation, the media flow was stopped for 1 h to allow initial attachment followed by continuous media flow with a flow rate of 6 mL/h. The biofilm reactor had a pseudo-steady state operation for 3 days and the average wet biomass generation rate was ∼0.03 g/day/reactor.

### ^13^C-Fingerprinting Amino Acids to Trace Flux Distributions

For labeled experiments, 20 mM [1,2-^13^C] glucose was used for cultivating both PAO1 and its c-di-GMP transconjugants, while [3-^13^C] sodium lactate was used for cultivating MR-1 and its c-di-GMP transconjugant. In planktonic mode, pseudo-steady-state shake flask cultures were harvested by centrifugation during mid-exponential phases. Cell pellets and supernatant were stored at −20°C before further analysis. For biofilm mode, ^13^C-labeled biomass in the tubular reactor was squeezed out for amino acid analysis. For labeled experiments, substrate concentrations (including glucose, lactate, and acetate) were measured using HPLC (Sivakumar et al., 2014). EPS formation was also determined (Jiao et al., 2010). To analyze proteinogenic amino acids, biomass pellets were hydrolyzed by 6 M HCl at 100°C, then air-dried and derivatized with *N*-*tert*-butyldimethylsilyl-*N*-methyltrifluoroacetamide (TBDMS) prior to GC-MS measurement (You et al., 2012). A published software was used to correct MS peaks (i.e., [M-57] and [M-159]) (Wahl et al., 2004). Mass isotopomer distributions (MID) (M0, M1, M2...) represent fragments with (0, 1, 2…) labeled carbons in amino acids. Due to overlapping peaks or product degradation, proline, arginine, cysteine, and tryptophan were not analyzed (Antoniewicz et al., 2007).

### Biofilm Imaging and Viability Analysis

Fresh PAO1 cells were grown on glass slides (1 cm^2^) for biofilm imaging and viability analyses. Briefly, PAO1 overnight cultures were used to inoculate Petri dishes containing M9 medium supplemented with 1 g/L unlabeled glucose. Biofilm cultures were incubated for 96 h (replacing spent medium with fresh M9 medium containing 1 g/L glucose every 48 h). After washing with PBS buffer, glass slides with attached biofilms (thickness 40∼60 μm) were transferred into new Petri dishes containing fresh M9 medium. To observe the attachment/settlement of planktonic cells on biofilms, biofilm cells were stained using SYTO 9 green fluorescent nucleic acid stain (Thermo Fisher Scientific, Waltham, MA, United States), then PAO1 planktonic cells (OD_600_ 0.7∼0.8) stained by orange dye Alexa Fluor 555 (Thermo Fisher Scientific, Waltham, MA, United States) were added into Petri dishes and incubated with biofilm slides for 1 h. The resulting biofilm was imaged using an Axio Imager M1 fluorescence microscope (Carl Zeiss, Inc., Germany) (note: the green color represents biofilm cells and orange color represents planktonic cells settled on slide surface). For parallel samples, live/dead staining images of PAO1 biofilm were also collected, where biofilm slides were stained with SYTO 9 (green) and propidium iodide (red) for 15 min at the room temperature before imaging (note: green stains all cells; while red indicates DNA in dead cells or extracellular DNA).

### Comparison of Glucose Catabolic Rates in PAO1 Cultures Using Dynamic ^13^C Labeling

**FIGURE 2:**
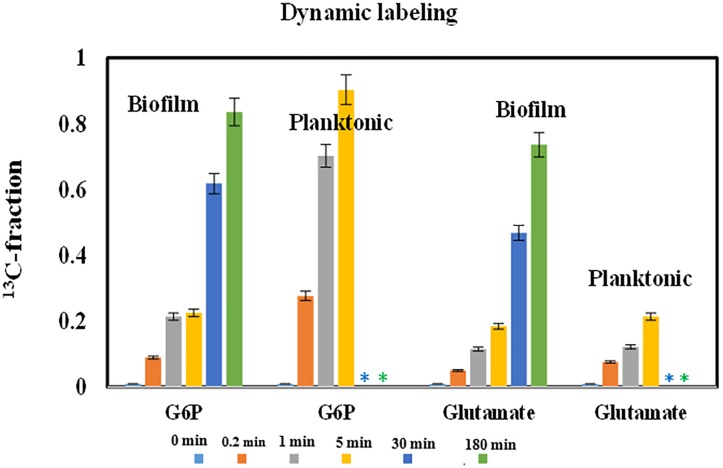
^13^C-fractions in starting and end point metabolites from central pathways for PAO1 biofilm and planktonic cells after pulse fully labeled glucose. The bar plots showed the increase of metabolite labeling as the function of time. Error bars are standard deviations of biological duplicates. ^∗^Long-time data points were not measured for planktonic cultures.

Glucose uptake in planktonic and biofilm PAO1 were measured by tracking ^13^C incorporation rates of two key metabolites (glucose-6-P and glutamate) after pulsing fully labeled glucose into unlabeled cultures at the room temperature. For planktonic ^13^C-experiments, PAO1 was grown in shake flasks with 1 g/L unlabeled glucose. Once cells reached late exponential phase (OD_600_ 0.7∼0.8) and ∼90% non-labeled glucose was consumed, fully labeled ^13^C glucose was added into the culture with final concentration of 2 g/L. After ^13^C-glucose additions, 15 mL of cell cultures were harvested by mixing cultures with 5 mL pre-cold M9-ice solutions at four sampling points (0, 0.2, 1, and 5 min). The samples were further quenched with ethanol-dry ice bath (−70°C) to reduce culture temperature to ∼0°C. Samples (with ice particles) were centrifuged at 8,000 rpm for 1 min and the pellets were kept at −20°C before LC–MS measurement. For dynamic ^13^C-experiments on biofilm, fresh PAO1 biofilm cells were prepared using glass slides (same as that for cell imaging). Before labeling experiments, glass slides with fresh unlabeled biofilm cells were washed by phosphate-buffered saline (PBS, 1X) buffer then soaked in 25 mL M9 medium containing 1 g/L fully labeled ^13^C-glucose for 0.2, 1, 5, 30, and 180 min. To harvest time-course samples, glass slides were placed in PBS-ice solution to quench cell metabolisms. Free metabolites were measured by LC–MS (Hollinshead et al., 2016). Briefly, quenched planktonic or biofilm cells were placed in cold methanol/chloroform solution (7:3 v/v) and shaken at 150 rpm at 4°C overnight. Deionized water was added to the solvent mix to extract cell metabolites. The aqueous phase was filtered through an Amicon Ultra centrifuge filter (3000 Da; EMD Millipore, Billerica, MA, United States) then lyophilized. The dried samples were dissolved in acetonitrile and water (6:4, v/v) solution for LC–MS analysis (Agilent Technologies 1200 Series equipped with a SeQuant Zic-pHILIC column) to determine MS distributions of targeted metabolites (Figure 2).

### Gene Expressions in Fresh Biofilm Cells

The qPCR was used to compare the expressions of glycolytic pathway genes between fresh biofilm cells from glass slides and planktonic cells. The protocol has been reported in our previous research (Choudhary et al., 2015). Generally, the cDNA was synthesized from the isolated RNA samples of PAO1 planktonic cells and glass slide biofilms using iScript cDNA Synthesis Kit (Biorad, Hercules, CA, United States). The primers were designed within primer blast (NCBI). The qPCR samples were prepared by mixing cDNA, primers, and iTaqTM universal SYBR Green Supermix (Biorad, Hercules, CA, United States). The qPCR reactions were accomplished with an Eppendorf Mastercycler Realplex thermal cycler (Eppendorf, Hauppauge, NY, United States). The condition of qPCR reactions was: heat activation at 95°C for 1 min, 40 cycles of denaturation at 95°C for 10 s, and annealing/extension at 60°C for 1 min. The melting curve was set at 95°C for 30 s, 45°C for 30 s, 20 min hold with temperature gradient, and 95°C for 1 min. The relative expression ratios of the selected genes were analyzed using the LinRegPCR program (Heart Failure Research Center, Netherlands) and equation below (Pfaffl, 2001):

$$\log_{2}\mathrm{Ratio}=\log_{2}[\frac{E_{T\;\arg\;\mathrm{et}}^{\Delta C_{q}^{T\;\arg\;\mathrm{et}}(\mathrm{planktonic}-\mathrm{Biofilm})}}{E_{\mathrm{Reference}}^{\Delta C_{q}^{\mathrm{Reference}}(\mathrm{planktonic}-\mathrm{Biofilm})}}]$$

**FIGURE 3:**
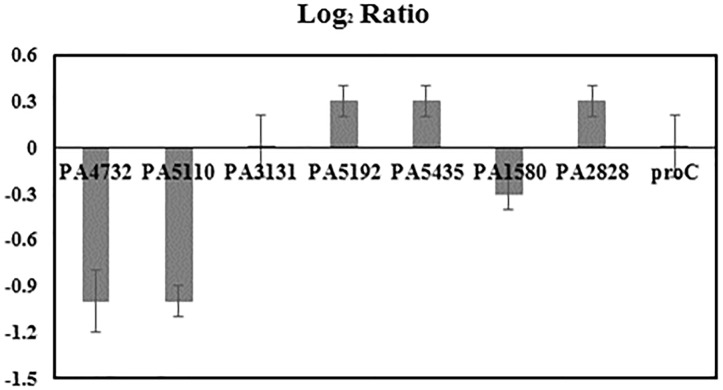
Expression fold change log_2_ ratio of selected PAO1 genes, including PA4732 (*pgi* Glucose-6-phosphate isomerase), PA5110 (*fbp* Fructose-1,6-bisphosphatase), PA3131 (*eda* Aldolase), PA5192 (*pckA* Phosphoenolpyruvate carboxykinase), PA5435 (*oadA* Transcarboxylase subunit), PA1580 (*gltA* Citrate synthase), PA2828 (probable *aminotransferase*), *proC* (housekeeping Pyrroline-5-carboxylate reductase) in biofilm cells compared to planktonic cells. Each target gene was tested in triplicate. A housekeeping gene *proC* was expressed at constant level in *Pseudomonas aeruginosa* PAO1 and thus used as the reference gene for the quantification of relative expression ratio of target genes (Pfaffl, 2001; Savli et al., 2003; Pan et al., 2012).

Δ*Cq* represented the difference in value of quantitation cycle between planktonic and biofilm samples. *E* described the qPCR efficiency. Both Δ*Cq* and *E* were calculated by the LinReg PCR program based on the raw data of qPCR experiments. The target samples were seven selected genes (PA4732, PA5110, PA3131, PA5192, PA5435, PA1580, and PA2828) related to glucose metabolism of *P. aeruginosa* (Figure 3). The reference sample was housekeeping gene *proC*(Savli et al., 2003).

### Metabolic Flux Analysis of Planktonic Culture and Tubular Reactor Biofilm Cells

^13^C-MFA was performed based on isotopomer data from proteinogenic amino acids from biofilm reactor and shake flask cultures (Supplementary Material). The software WUflux (He et al., 2016) was used for flux calculations. Biomass composition was modified based on previous study (Bartell et al., 2016). The MFA model included the EMP (Embden-Meyerhof-Parnas) pathway, the OPP (oxidative pentose phosphate) pathway, the ED (Entner-Doudoroff) pathway, the TCA cycle, the glyoxylate shunt, and biomass synthesis (Stover et al., 2000). Based on KEGG database, PAO1 contains fructose-1,6-bisphosphatase but lacks phosphofructokinase and thus the reaction (F6P→FBP) was deleted from the model. Since the precise measurement of actual glucose utilization for biofilm production was very difficult due to the presence of both planktonic cells and biofilm cells in tubular reactors, ^13^C-MFA profiled relative fluxes by assuming glucose uptake rate as 100 units. The relative fluxes were solved by minimizing a quadratic error function that calculated the differences between predicted and measured isotopomer patterns (*n* = 2). The confidence intervals of fluxes were estimated as following. The model randomly perturbed both biomass equation for EPS formations by ± 10% and amino acid MID data within measurement standard deviations for 500 times to simulate experimental uncertainty. Based on each new dataset, the model re-calculated fluxes. Then confident intervals were estimated based on the variations of resulting fluxes (He et al., 2016).

## Results

### Dynamic Labeling of Free Metabolites in Planktonic and Biofilm Cells

Some bacterial species favor the growth on solid surfaces, while others favor planktonic mode. Comparisons between biofilm and planktonic cell growths have been extensively reported (Heffernan et al., 2009). To understand overall PAO1 biofilm physiologies, we prepared fresh PAO1 biofilms on glass slides. Before pulsing ^13^C-glucose for the dynamic labeling of biofilm cells, we washed glass slides to remove planktonic cells attached on the biofilm surface. Fluorescence microscope imaging confirmed that few planktonic cells (pre-labeled with orange dye) remained on the biofilm surface (in green color) (Figure 1). These biofilm cells on glass slides could be easily sampled and quenched for fast turnover metabolite analysis or cell imaging. Here, dynamic labeling technique was used to measure metabolite turnover rates in biofilm cells from glass slides, which were then compared with shake flask cultures. Figure 2 showed labeling rates for two key metabolites after ^13^C-glucose was pulsed into biofilm or planktonic cells. As expected, labeling rates of G6P (first metabolic node after glucose uptake) for planktonic cells were much faster than biofilm cells, and the ^13^C enrichment reached saturation within 5 min. However, it took 180 min for G6P labeling to reach saturation in biofilm cells. Interestingly, final labeling percentages of G6P reached > 85% in biofilm cells, indicating that the majority of biofilm cells were metabolically active for glucose utilizations despite the slow rate. Spatial stratification of oxygen and glucose within the biofilm was a possible explanation. Moreover, free glutamate (the key downstream product from the TCA cycle for biomass synthesis) from both planktonic cells and biofilm cells were labeled much slower than that of G6P (20∼25% after 5 min). This observation could be explained by the fact that metabolite turnover rates in amino acid synthesis pathways were much slower than the glucose uptake rates under both biofilm and planktonic modes.

### Fluxomes of Planktonic and Biofilm *Pseudomonas* Cells

**FIGURE 4:**
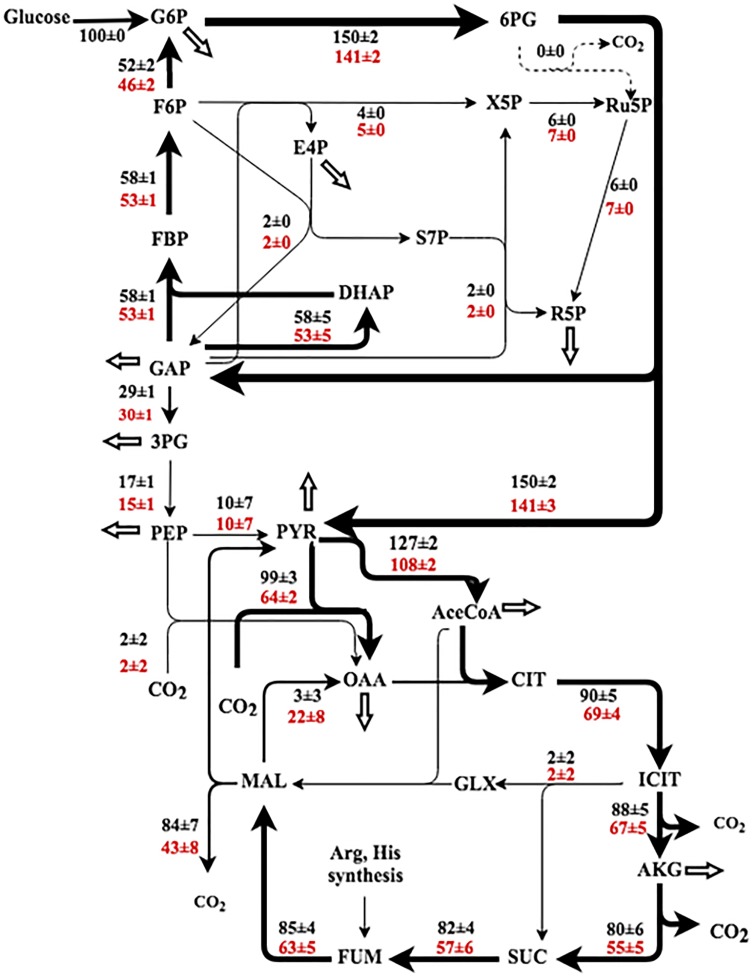
Flux ratio of *Pseudomonas aeruginosa* as planktonic (black) and biofilm (red). The fluxes were normalized to the glucose uptake rate (represented as 100), and the fluxes are represented as ‘best fit ± confidence intervals’ based on the measured isotopomer distributions (biological duplicates). The arrow thickness relates to the magnitude of flux. The white arrows represent the fluxes toward biomass synthesis. 3PG, 3-phosphoglycerate; 6PG, 6-phosphogluconate; AceCoA, acetyl-CoA; DHAP, dihydroxyacetone phosphate; E4P, erythrose 4-phosphate; FBP, fructose 1,6-bisphosphate; F6P, fructose 6-phosphate; G6P, glucose 6-phosphate; GAP, glyceraldehyde 3-phosphate; GLX, glyoxylate; ICT, isocitrate; MA L, malate; OAA, oxaloacetate; PEP, phosphoenolpyruvate; PYR, pyruvate; R5P, ribose 5-phosphate; Ru5P, ribulose-5-phosphate; RuBP, ribulose-1,5-diphosphate; S7P, sedoheptulose-7-phosphate; SUC, succinate; X5P, xylulose-5-phosphate.

Planktonic fluxes in *P. aeruginosa* have been reported (Berger et al., 2014; Lassek et al., 2016; Opperman and Shachar-Hill, 2016). These studies highlighted the glucokinase (phosphorylate glucose to G6P then to 6PG) and ED pathways that are mainly responsible for glucose catabolism. The magnitude of fluxes through the oxidative pentose phosphate pathway, glyoxylate shunt, and the TCA cycle varied among different reports. This study examined the *P. aeruginosa* metabolism in both planktonic and biofilm modes at the room temperature. By cultivation with [1, 2-^13^C] labeled glucose in tubular reactors, the resulting proteinogenic amino acids stored labeling information (i.e., ^13^C-fingerprinting) that could be used for ^13^C-MFA. In contrast to dynamic labeling experiments (i.e., G6P turnover rates) that showed overall metabolic rates in the biofilm were much slower than their free-floating counterparts, metabolic flux distributions had smaller variations between biofilm and planktonic modes after normalizing glucose uptake as 100%. Most flux values in biofilm cells differed within 20% compared to planktonic cells. For both cultivation modes, flux network showed a complete carbohydrate degradation loop: Entner-Doudoroff-Embden-Meyerhof-Parnas (EDEMP) cycle (G6P→6PG→GAP→F6P→G6P) (Figure 4), possibly due to metabolic congestion at the lower segment of glycolysis. Compared to biofilm cells, planktonic cells had moderately higher fluxes through the TCA cycle. Similar EDEMP cycle has been observed in *Pseudomonas putida* (Nikel et al., 2015). *Pseudomonas* is well-known for using the ED pathway rather than the EMP for the glucose catabolism due to the absence of phosphofructokinase (Berger et al., 2014). The ED pathway is not beneficial to ATP generation, but it reduces metabolic cost for enzyme synthesis (Stettner and Segrè, 2013). More importantly, the formation of EDEMP cycle could improve NADPH generation to diminish oxidative stress and to promote the biosynthesis of C6 sugar phosphates (the precursor of EPS). The TCA cycle in *Pseudomonas* species was reported to operate with the pyruvate shunt, which was catalyzed by malic enzyme and pyruvate carboxylase (malate→pyruvate→OAA) (Fuhrer et al., 2005; del Castillo et al., 2007). Same pyruvate shunt was observed in both PAO1 planktonic and biofilm cells (Figure 4). For example, very little malate dehydrogenase flux was observed in planktonic cells, and a significant amount of OAA was synthesized from pyruvate. The pyruvate shunt coupled with other anaplerotic pathways (including glyoxylate shunt) could regulate fluxes between glycolysis nodes (PEP and Pyruvate) and the TCA nodes (Malate and OAA) to increase flux network plasticity.

The variations of flux network between planktonic and biofilm cells were further investigated via qPCR analysis. We compared the expression levels of seven key genes related to glucose metabolism (including *pgi*, *fbp*, *edaB*, *pckA*, *oadA*, *gltA*, *aminotransferase*) along with the housekeeping gene *proC*. According to the results shown in Figure 3, *proC* was not differentially expressed under the two culture modes, which was consistent with a previous research (Savli et al., 2003). The qPCR results also indicated that expression levels of all selected genes between biofilm and planktonic samples have relatively small differences (note: less than twofold). This result, though incomplete to reflect global genetic regulations, suggested PAO1 could maintain normal functions of many central genes for glucose catabolism during the active biofilm growth phase.

### ^13^C Fingerprinting of the PAO1 Transconjugant and *Shewanella* Under Planktonic and Biofilm Conditions

**FIGURE 5:**
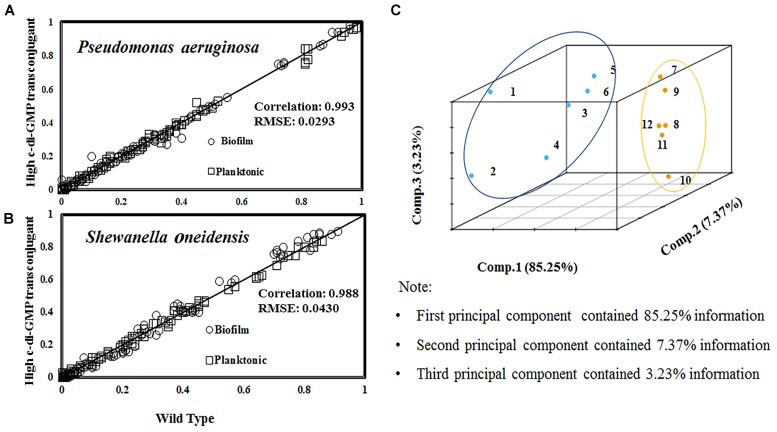
Correlation of amino acids labeling between wild type and c-di-GMP transconjugants (represented by R^2^). The RMSE was calculated based on the variations of amino acid labeling data between planktonic and biofilm samples from the same strain. **(A)** Comparison of MID from PAO1 and high c-di-GMP transconjugant samples under planktonic and biofilm modes (grown with [1,2-^13^C] glucose). **(B)** Comparison of MID from MR-1/high c-di-GMP transconjugant samples under planktonic and biofilm modes (grown with [3-^13^C] lactate). **(C)** Principal component analysis (PCA) of amino acid labeling for 12 conditions. **Condition 1∼6 for MR-1**: (1) planktonic cells at the mid-log phase; (2) high c-di-GMP transconjugant planktonic cells at the mid-log phase; (3) MR-1 in tubular biofilm reactor at day 3; (4) MR-1 in tubular biofilm reactor at day 4; (5) high c-di-GMP transconjugant in tubular biofilm reactor at day 3; (6) high c-di-GMP transconjugant in tubular biofilm reactor at day 4. **Condition 7∼12 for PAO1**: (7) planktonic cells at the mid-log phase; (8) high c-di-GMP transconjugant planktonic cells at the mid-log phase; (9) low c-di-GMP planktonic cells at the mid-log phase; (10) PAO1 in the tubular biofilm reactor; (11) high c-di-GMP transconjugant in the tubular biofilm reactor; (12) low c-di-GMP transconjugant in the tubular biofilm reactor. Principal Component Analysis was generated by R (version 3.2.2) for 12 biofilm/planktonic conditions. Principal components PC1, PC2 and PC3 were included in this study.

We examined the transconjugants (i.e., high or low c-di-GMP expressions) of PAO1 via ^13^C-labeling of proteinogenic amino acids from tubular reactors or shake flask cultures. The ^13^C-fingerprints (MID of amino acid labeling) of PAO1 and high c-di-GMP transconjugant were collected from planktonic cultures and biofilm reactors and plotted in Figure 5A. Compared to PAO1 wild type, its high c-di-GMP transconjugant produced 1.9-fold more EPS and twice more biofilm in tubular reactors. Moreover, using labeling data of PAO1 planktonic culture as the baseline, MID data were found to have high correlations (*R*^2^ = 0.99) correlations between PAO1 and its transconjugant samples from planktonic and biofilm cultures (Figure 5A). This observation inferred that the mutant and the wild type shared similar flux distributions (i.e., change of planktonic or biofilm growth rate does not require significant intracellular flux rewiring). To obtain a broader understanding of flux regulations, similar ^13^C-fingerpring experiments on *S. oneidensis* MR-1 and its high c-di-GMP transconjugant were performed. The MID of proteinogenic amino acids also demonstrated strong correlations (*R*^2^ = 0.99) among MR-1 and its c-di-GMP transconjugant (Figure 5B). However, the root-mean-square error (RMSE) of labeling data variations between planktonic and biofilm cells in MR-1 was 1.5-fold higher than the RMSE obtained from PAO1 cultures (Figure 5A). Further principal component analysis (PCA) examined MID (as the features) of amino acids from different ^13^C-cultures (planktonic or biofilm cultures of PAO1, MR-1, and their transconjugants) (Figure 5C). Both the RMSE and PCA results indicated that the MR-1 metabolism could be more affected by its biofilm growth mode than the PAO1. This observation (i.e., MR-1 flux network was more flexible) was consistent to the reproted versatility of MR-1 metabolisms (Guo et al., 2015). For example, O_2_ conditions could influence acetate overflows and intracellular fluxome in MR-1 (Tang et al., 2007). Nevertheless, different bacteria may have different capabilities for minimizing the change of flux network when cells switch from planktonic to biofilm growth.

## Discussion

**FIGURE 6:**
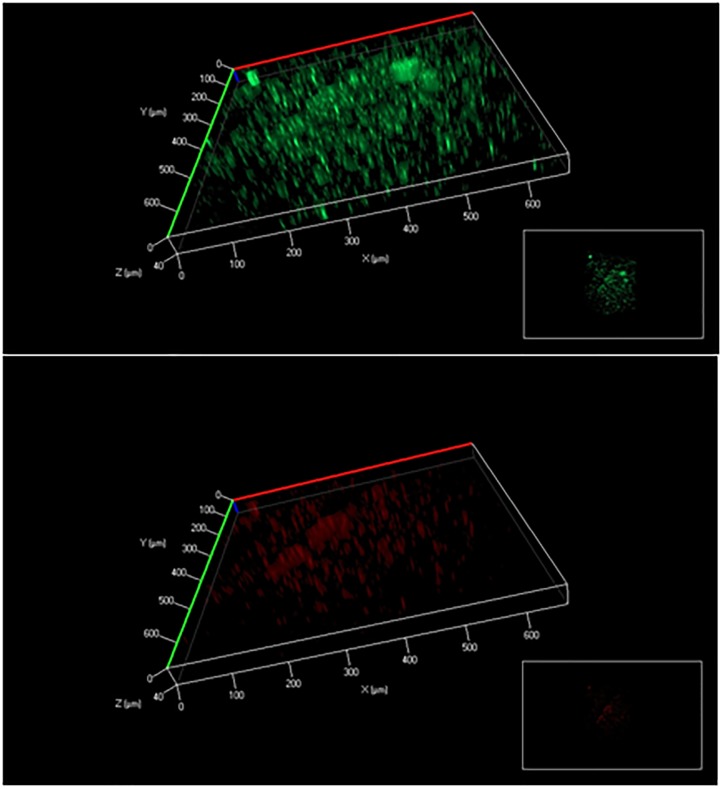
Biofilm cell viability analysis (above: all cells; below: dead cells). The biofilm thickness was about 40∼60 μm.

There is a consensus that cell attachment onto surfaces strongly influences microbial metabolism. For example, *P. aeruginosa* displays phenotypic changes during biofilm development (Sauer et al., 2002). Because of temporal and structural variations, conflicting observations have been reported on biofilm growth kinetics and metabolic activities compared to free-floating cells (van Loosdrecht et al., 1990; Heffernan et al., 2009). In this study, glucose uptake by fresh biofilm cells (based on G6P labeling) was found to be much slower than planktonic cells, while both planktonic and biofilm cells had sluggish glutamate synthesis (Figure 2). Moreover, biofilm cells employed a relatively similar flux network as planktonic cultures: PAO1 glucose catabolism was mainly dependent on the EDEMP/TCA loops, pyruvate shunt, and several anaplerotic pathways. Meanwhile, expression levels of essential genes in PAO1 central pathways were analyzed and no target gene in glucose catabolism was highly up-regulated or down-regulated (Log2 ratio of 2 as the cutoff, Figure 3) between planktonic and fresh biofilm cells (note: only two genes in glycolysis, *pgi* and *fbp* appeared to be moderately repressed in biofilms compared to planktonic cells). The gene expressions in fresh biofilm cells indicated that their metabolism could maintain stable catabolic functions. These biofilm metabolic features could be explained by three reasons. First, our cultivations offered optimal biofilm growth and minimized biofilm heterogeneity. For example, majority of cells were alive in the freshly prepared biofilm (thickness of only 40∼60 μm) on glass slides (dominating green signals compared to red signals in Figure 6), while the use of silicone tubing bioreactor improved oxygen and nutrient transports for biofilm biomass generation. Second, cells located in the peripheral layers of biofilms might contribute significantly to biofilm growth since these cells received nutrients at a level similar to that of planktonic cells. Third, bacterial metabolism inherently demonstrated robust ratios for resource allocations.

In a broader perspective, bacterial flux networks are not straightforwardly correlated with gene expressions (Chubukov et al., 2013) or proteomic profiles (Lassek et al., 2016). Although bacterial physiologies are sensitive to nutrient and growth conditions, flux ratios/network may demonstrate small perturbations or certain rigidity against genetic and environmental changes (Fischer and Sauer, 2005). For example, bacterial flux distribution under salt stresses could remain the same as normal growth conditions, which was in stark contrast to slower growth rate and high changes of transcript profiles (Tang et al., 2009). The conservation of microbial fluxomics (i.e., metabolic robustness) is regarded as the principle of how cell metabolism distributes resources for biomass growth, while microbial species may demonstrate different degrees of flux conservations during their biofilm growth.

The methods and observations in this study still have limitations. First, the variation in growth conditions and surface materials from different lab cultures may influence cell metabolisms. Second, a biofilm culture includes at least three sub-populations (planktonic cells, fast growing biofilm cells, and dormant/dead biofilm cells in deep layers, as shown in Figure 6). ^13^C-fingerprining of proteinogenic amino acids could only track these actively growing cells (i.e., on the top of the biofilms or deposited from planktonic phase) that consumed major nutrient resources for biomass synthesis. This approach failed to provide unique insights into the metabolic topology or flux network plasticity for these dormant/slow-growth biofilm cells under environmental stresses. To further reveal metabolic activities in heterogeneous biofilm, new tools (such as population snapshot measurements by cell sorting) are required to integrate with ^13^C-labeling techniques. Some cell patterning technologies may also be adapted to obtain biofilms with well-defined structures (thus reduced heterogeneity) to allow better understanding of biofilm metabolism (Ren et al., 2012, 2013a,b; Gu et al., 2013). This is part of our ongoing work.

## Conclusion

The flux network in biofilm cell is not yet well understood. This study elucidated metabolic features of PAO1 biofilm cells via comparative ^13^C labeling. Bacterial cells within biofilms differ in physiologies because of nutrient and oxygen limitations, but biofilm flux distributions could still show certain degree of invariability. Specifically, PAO1 cells could fairly maintain its flux distributions and gene expressions as its planktonic culture during active biofilm development. To further decipher biofilm metabolism and regulations in different bacterial sepcies, our future work aims to expand metabolite coverage as well as spatial and temporal anlaysis of biofilm subpopulations.

## Availability of Data and Materials

All isotopomer data and metabolic reactions are included as supporting information.

## Author Contributions

YT, DR, and BC initiated the project and designed experiments. NW, HW, CN, and MM performed experiments and modeling analysis. All authors wrote and approved the final version of the manuscript.

## Conflict of Interest Statement

The authors declare that the research was conducted in the absence of any commercial or financial relationships that could be construed as a potential conflict of interest.
