# Supplementary material for: Bacterial Metabolism During Biofilm Growth Investigated by 13C Tracing
Source: Front Microbiol. 2018 Nov 20;9:2657. doi: 10.3389/fmicb.2018.02657 (PMC6255981; doi:10.3389/fmicb.2018.02657)
Supplement: Supplementary file 1 [file Data_Sheet_1.PDF]

Supporting Information

Isotopomer data for *Shewanella oneidensis* MR-1 WT P (wild type planktonic cells), MR-1 PYEDQ P (biofilm-overexpressing planktonic cells), MR-1 WT B3 (wild type biofilm 3-days cells), MR-1 WT B4 (wild type biofilm 4-days cells), MR-1 PYEDQ B3 (biofilm-overexpressing biofilm 3-days cells), MR-1 PYEDQ B4 (biofilm-overexpressing biofilm 4-days cells).

Biofilm-overexpressing = high c-di-GMP; Biofilm-downregulated = low c-di-GMP

			**WT P**	**PYEDQ P**	**WT B3**	**WT B4**	**PYEDQ B3**	**PYEDQ B4**
alanine	M-57	M0	0.02±0.01	0.03±0.01	0.02±0.01	0.02±0.00	0.02±0.00	0.02±0.00
		M1	0.84±0.02	0.84±0.02	0.92±0.01	0.91±0.01	0.90±0.01	0.92±0.01
		M2	0.1±0.00	0.1±0.00	0.06±0.01	0.06±0.00	0.06±0.00	0.05±0.00
		M3	0.04±0.01	0.03±0.01	0.01±0.00	0.01±0.01	0.01±0.01	0.01±0.01
	M-159	M0	0.03±0.01	0.03±0.01	0.02±0.01	0.02±0.01	0.03±0.01	0.03±0.01
		M1	0.86±0.02	0.84±0.01	0.9±0.02	0.89±0.02	0.88±0.02	0.9±0.02
		M2	0.10±0.01	0.12±0.01	0.08±0.01	0.08±0.01	0.09±0.01	0.08±0.01
glycine	M-57	M0	0.71±0.01	0.69±0.01	0.78±0.01	0.73±0.01	0.8±0.01	0.79±0.02
		M1	0.22±0.01	0.23±0.01	0.18±0.02	0.21±0.01	0.17±0.02	0.18±0.01
		M2	0.08±0.01	0.08±0.01	0.04±0.01	0.06±0.01	0.04±0.01	0.04±0.0
	M-85	M0	0.77±0.01	0.75±0.01	0.82±0.01	0.78±0.02	0.84±0.01	0.82±0.01
		M1	0.23±0.01	0.25±0.01	0.18±0.01	0.22±0.01	0.16±0.01	0.18±0.01
valine	M-57	M0	0±0.00	0±0.00	0.01±0.00	0.01±0.00	0±0.00	0.01±0.00
		M1	0.01±0.00	0.02±0.00	0.04±0.02	0.02±0.01	0.03±0.01	0.03±0.01
		M2	0.83±0.02	0.8±0.02	0.81±0.02	0.85±0.02	0.88±0.02	0.86±0.02
		M3	0.13±0.01	0.13±0.01	0.11±0.01	0.11±0.03	0.07±0.03	0.1±0.04
		M4	0.03±0.01	0.05±0.00	0.03±0.00	0.02±0.00	0.01±0.00	0±0.00
		M5	0±0.00	0±0.00	0±0.00	0±0.00	0.01±0.00	0±0.00
	M-159	M0	0.01±0.00	0±0.00	0.01±0.00	0.01±0.00	0.01±0.00	0.01±0.00
		M1	0.03±0.00	0.03±0.00	0.03±0.00	0.03±0.00	0.02±0.00	0.02±0.00
		M2	0.83±0.01	0.83±0.01	0.82±0.02	0.85±0.02	0.89±0.02	0.87±0.01
		M3	0.13±0.01	0.12±0.01	0.13±0.01	0.09±0.01	0.08±0.01	0.09±0.01
		M4	0.01±0.00	0.01±0.00	0.01±0.00	0.02±0.00	0.01±0.00	0.01±0.00
leucine	M-15	M0	0±0.00	0±0.00	0±0.00	0±0.00	0±0.00	0±0.00
		M1	0±0.00	0±0.00	0±0.00	0±0.00	0±0.00	0±0.00
		M2	0.2±0.01	0.19±0.01	0.18±0.01	0.19±0.01	0.2±0.01	0.19±0.01
		M3	0.73±0.02	0.74±0.02	0.73±0.02	0.72±0.01	0.73±0.02	0.72±0.02
		M4	0.06±0.01	0.06±0.01	0.06±0.01	0.06±0.01	0.06±0.01	0.06±0.01
		M5	0.02±0.01	0.01±0.01	0.03±0.01	0.03±0.01	0.02±0.01	0.03±0.01
		M6	0±0.00	0±0.00	0±0.00	0±0.00	0±0.00	0±0.00
	M-159	M0	0.01±0.00	0±0.00	0±0.00	0±0.00	0.01±0.00	0±0.00
		M1	0.01±0.00	0.01±0.00	0.01±0.00	0.01±0.00	0.01±0.00	0.01±0.00
		M2	0.05±0.01	0.05±0.01	0.04±0.0	0.05±0.0	0.05±0.0	0.05±0.0
		M3	0.78±0.02	0.8±0.01	0.84±0.02	0.82±0.02	0.78±0.02	0.85±0.02
		M4	0.12±0.01	0.11±0.01	0.07±0.01	0.07±0.01	0.12±0.01	0.06±0.01
		M5	0.03±0.0	0.03±0.0	0.04±0.00	0.05±0.01	0.03±0.00	0.04±0.01
isoleucine	M-15	M0	0±0.00	0±0.00	0±0.00	0.01±0.00	0±0.00	0±0.00
		M1	0.01±0.00	0.01±0.00	0.01±0.00	0.02±0.01	0.02±0.01	0.02±0.00
		M2	0.23±0.02	0.21±0.02	0.28±0.02	0.25±0.02	0.30±0.02	0.30±0.01
		M3	0.37±0.02	0.41±0.02	0.42±0.03	0.41±0.03	0.4±0.02	0.40±0.02
		M4	0.35±0.01	0.31±0.02	0.25±0.01	0.29±0.01	0.27±0.01	0.26±0.02
		M5	0.05±0.01	0.05±0.01	0.02±0.01	0.02±0.01	0.02±0.01	0.02±0.01
		M6	0±0.00	0±0.00	0±0.00	0±0.00	0±0.00	0±0.00
	M-159	M0	0±0.00	0±0.00	0±0.00	0±0.00	0±0.00	0±0.00
		M1	0.01±0.00	0.01±0.00	0.01±0.00	0.01±0.00	0.01±0.00	0.01±0.00
		M2	0.33±0.01	0.35±0.01	0.41±0.01	0.37±0.01	0.41±0.01	0.42±0.01

		M3	0.45±0.01	0.44±0.02	0.42±0.01	0.43±0.01	0.42±0.01	0.42±0.01
		M4	0.2±0.01	0.18±0.01	0.15±0.01	0.17±0.01	0.15±0.01	0.15±0.01
		M5	0.01±0.00	0.01±0.00	0.01±0.00	0.01±0.00	0 ±0.00	0.01±0.00
serine	M-57	M0	0.05±0.01	0.05±0.01	0.06±0.01	0.06±0.01	0.06±0.01	0.06±0.01
		M1	0.75±0.02	0.75±0.02	0.83±0.02	0.81±0.02	0.86±0.02	0.85±0.02
		M2	0.15±0.01	0.15±0.01	0.09±0.01	0.1±0.01	0.07±0.01	0.08±0.01
		M3	0.05±0.01	0.05±0.01	0.02±0.01	0.03±0.0	0.01±0.00	0.02±0.01
	M-159	M0	0.06±0.01	0.06±0.01	0.06±0.01	0.07±0.01	0.06±0.01	0.06±0.01
		M1	0.80±0.02	0.8±0.01	0.85±0.02	0.83±0.01	0.88±0.02	0.87±0.02
		M2	0.15±0.02	0.14±0.01	0.08±0.01	0.1±0.01	0.06±0.01	0.08±0.01
threonine	M-57	M0	0.08±0.01	0.08±0.03	0±0.00	0±0.00	0±0.00	0±0.00
		M1	0.24±0.03	0.2±0.03	0.3±0.03	0.27±0.03	0.32±0.03	0.32±0.03
		M2	0.38±0.03	0.38±0.03	0.41±0.03	0.4±0.03	0.41±0.03	0.41±0.03
		M3	0.30±0.01	0.33±0.03	0.28±0.02	031±0.02	0.26±0.02	0.26±0.02
		M4	0±0.00	0.03±0.00	0.01±0.00	0.01±0.00	0±0.00	0.01±0.00
	M-159	M0	0.06±0.01	0.07±0.02	0.01±0.01	0.02±0.0	0.02±0.01	0.02±0.0
		M1	0.36±0.02	0.35±0.02	0.41±0.02	0.39±0.02	0.44±0.02	0.44±0.02
		M2	0.42±0.03	0.43±0.03	0.43±0.02	0.43±0.02	0.41±0.02	0.41±0.02
		M3	0.15±0.01	0.16±0.01	0.14±0.01	0.16±0.01	0.13±0.01	0.14±0.01
phenylalanine	M-57	M0	0±0.00	0±0.00	0±0.00	0±0.00	0±0.00	0±0.00
		M1	0±0.00	0±0.00	0±0.00	0±0.00	0±0.00	0±0.00
		M2	0.02±0.01	0.02±0.01	0.03±0.01	0.03±0.01	0.03±0.01	0.03±0.01
		M3	0.64±0.03	0.61±0.03	0.74±0.03	0.71±0.03	0.77±0.03	0.75±0.03
		M4	0.23±0.02	0.25±0.02	0.18±0.02	0.2±0.03	0.16±0.02	0.18±0.02
		M5	0.08±0.01	0.08±0.01	0.04±0.01	0.04±0.01	0.03±0.01	0.04±0.01
		M6	0.02±0.00	0.02±0.00	0.01±0.00	0.01±0.00	0±0.00	0±0.00
		M7	0±0.00	0±0.00	0±0.00	0±0.00	0±0.00	0±0.00
		M8	0±0.00	0±0.00	0±0.00	0±0.00	0±0.00	0±0.00
		M9	0±0.00	0±0.00	0±0.00	0±0.00	0±0.00	0±0.00
	M-159	M0	0±0.00	0±0.00	0±0.00	0±0.00	0±0.00	0±0.00
		M1	0±0.00	0±0.00	0±0.00	0±0.00	0±0.00	0±0.00
		M2	0.02±0.00	0.03±0.00	0.03±0.00	0.03±0.00	0.03±0.00	0.03±0.00
		M3	0.66±0.01	0.63±0.01	0.75±0.02	0.71±0.02	0.78±0.02	0.76±0.02
		M4	0.24±0.01	0.25±0.01	0.18±0.01	0.20±0.01	0.16±0.01	0.18±0.01
		M5	0.06±0.01	0.07±0.01	0.03±0.01	0.04±0.00	0.02±0.01	0.03±0.01
		M6	0.01±0.00	0.02±0.00	0±0.00	0.01±0.00	0±0.00	0±0.00
		M7	0±0.00	0±0.00	0±0.00	0±0.00	0±0.00	0±0.00
		M8	0±0.00	0±0.00	0±0.00	0±0.00	0±0.00	0±0.00
aspartate	M-57	M0	0±0.00	0±0.00	0±0.00	0±0.00	0±0.00	0±0.00
		M1	0.21±0.01	0.22±0.01	0.3±0.01	0.27±0.01	0.3±0.01	0.33±0.01
		M2	0.4±0.01	0.4±0.02	0.41±0.01	0.39±0.03	0.41±0.01	0.4±0.01
		M3	0.35±0.01	0.33±0.01	0.29±0.01	0.32±0.03	0.29±0.01	0.26±0.01
		M4	0.03±0.0	0.03±0.0	0.01±0.0	0.02±0.0	0.01±0.0	0.01±0.0
	M-159	M0	0±0.00	0.01±0.00	0±0.00	0±0.00	0±0.00	0±0.00
		M1	0.34±0.01	0.34±0.01	0.42±0.01	0.39±0.01	0.42±0.01	0.45±0.01
		M2	0.47±0.01	0.46±0.01	0.43±0.01	0.44±0.01	0.43±0.01	0.42±0.01
		M3	0.2±0.01	0.2±0.01	0.15±0.01	0.17±0.01	0.15±0.01	0.14±0.01
glutamate	M-57	M0	0±0.00	0±0.00	0±0.00	0±0.00	0±0.00	0±0.00
		M1	0.01±0.00	0.01±0.00	0±0.00	0±0.00	0±0.00	0±0.00
		M2	0.31±0.01	0.33±0.01	0.42±0.01	0.38±0.01	0.45±0.01	0.44±0.01
		M3	0.45±0.01	0.45±0.01	0.41±0.01	0.44±0.01	0.41±0.01	0.41±0.01
		M4	0.21±0.01	0.2±0.01	0.15±0.01	0.18±0.01	0.14±0.01	0.14±0.01
		M5	0.01±0.00	0.01±0.00	0±0.00	0.01±0.00	0±0.00	0.01±0.00
	M-159	M0	0±0.00	0±0.00	0±0.00	0±0.00	0±0.00	0±0.00
		M1	0.02±0.00	0.02±0.00	0.03±0.00	0±0.00	0±0.00	0.03±0.00

		M2	0.45±0.01	0.47±0.01	0.54±0.01	0.52±0.01	0.59±0.01	0.56±0.01
		M3	0.47±0.01	0.47±0.01	0.4±0.01	0.45±0.01	0.4±0.01	0.4±0.01
		M4	0.04±0.00	0.04±0.00	0.02±0.00	0.02±0.00	0.01±0.00	0.01±0.00
lysine	M-57	M0	0±0.00	0±0.00	0±0.00	0±0.00	0±0.00	0±0.00
		M1	0.01±0.00	0.02±0.00	0.02±0.00	0.02±0.00	0.02±0.00	0.02±0.00
		M2	0.27±0.03	0.3±0.03	0.38±0.03	0.31±0.01	0.38±0.03	0.37±0.03
		M3	0.41±0.03	0.41±0.03	0.4±0.02	0.4±0.01	0.4±0.02	0.4±0.02
		M4	0.27±0.01	0.25±0.01	0.19±0.01	0.24±0.01	0.19±0.02	0.2±0.02
		M5	0.03±0.00	0.03±0.00	0±0.00	0.02±0.00	0±0.00	0.01±0.00
		M6	0±0.00	0±0.00	0±0.00	0±0.00	0±0.00	0±0.00
	M-159	M0	0.01±0.00	0.01±0.00	0.01±0.00	0.01±0.00	0.01±0.00	0.01±0.00
		M1	0.02±0.00	0.02±0.00	0.02±0.00	0.02±0.00	0.02±0.00	0.02±0.00
			0.33±0.01	0.35±0.01	0.4±0.01	0.37±0.01	0.44±0.01	0.43±0.01
			0.45±0.01	0.44±0.01	0.42±0.01	0.43±0.01	0.4±0.01	0.4±0.01
			0.19±0.01	0.18±0.01	0.15±0.01	0.17±0.01	0.13±0.01	0.14±0.0
			0.01±0.00	0.01±0.00	0.01±0.00	0.01±0.00	0±0.00	0±0.00
histidine	M-57	M0	0±0.00	0±0.00	0±0.00	0.01±0.00	0.01±0.00	0±0.00
		M1	0.04±0.00	0.04±0.00	0.04±0.00	0.04±0.00	0.04±0.00	0.04±0.00
		M2	0.26±0.02	0.27±0.02	0.17±0.01	0.28±0.01	0.28±0.01	0.29±0.01
		M3	0.57±0.02	0.56±0.02	0.6±0.01	0.57±0.02	0.6±0.01	0.59±0.01
		M4	0.11±0.02	0.1±0.01	0.08±0.01	0.08±0.0	0.7±0.01	0.07±0.01
		M5	0.02±0.00	0.02±0.00	0.01±0.00	0.02±0.0	0.01±0.00	0±0.00
		M6	0±0.00	0±0.00	0±0.00	0±0.00	0±0.00	0±0.00
	M-159	M0	0.04±0.00	0.04±0.00	0.04±0.00	0.04±0.00	0.04±0.00	0.04±0.00
		M1	0.25±0.01	0.25±0.01	0.26±0.01	0.26±0.02	0.26±0.02	0.27±0.01
		M2	0.55±0.01	0.54±0.01	0.58±0.01	0.56±0.02	0.58±0.02	0.58±0.01
		M3	0.11±0.01	0.12±0.01	0.09±0.01	0.1±0.01	0.08±0.01	0.08±0.01
		M4	0.03±0.00	0.04±0.00	0.02±0.00	0.03±0.00	0.02±0.00	0.02±0.00
		M5	0.01±0.00	0.01±0.00	0.01±0.00	0.02±0.00	0.02±0.00	0.02±0.00
tyrosine	M-57	M0	0±0.00	0±0.00	0±0.00	0±0.00	0±0.00	0±0.00
		M1	0±0.00	0±0.00	0±0.00	0±0.00	0±0.00	0.01±0.00
		M2	0.02±0.00	0.03±0.00	0±0.00	0±0.00	0.03±0.00	0.03±0.00
		M3	0.64±0.02	0.62±0.05	0.77±0.05	0.72±0.05	0.76±0.05	0.76±0.05
		M4	0.23±0.01	0.24±0.03	0.19±0.03	0.21±0.03	0.17±0.03	0.17±0.03
		M5	0.08±0.01	0.08±0.03	0.04±0.03	0.05±0.01	0.03±0.01	0.04±0.01
		M6	0.02±0.00	0.02±0.00	0.01±0.00	0.01±0.00	0±0.00	0±0.00
		M7	0±0.00	0±0.00	0±0.00	0±0.00	0±0.00	0±0.00
		M8	0±0.00	0±0.00	0±0.00	0±0.00	0±0.00	0±0.00
		M9	0±0.00	0±0.00	0±0.00	0±0.00	0±0.00	0±0.00
	M-159	M0	0±0.00	0±0.00	0±0.00	0±0.00	0±0.00	0±0.00
		M1	0.01±0.00	0.01±0.00	0.01±0.00	0.01±0.00	0.01±0.00	0.01±0.00
		M2	0.03±0.01	0.03±0.01	0.04±0.01	0.04±0.01	0.04±0.01	0.04±0.01
		M3	0.65±0.01	0.62±0.02	0.74±0.02	0.7±0.02	0.76±0.02	0.75±0.01
		M4	0.23±0.01	0.24±0.02	0.17±0.01	0.2±0.01	0.15±0.01	0.16±0.01
		M5	0.06±0.0	0.07±0.0	0.03±0.0	0.05±0.0	0.03±0.0	0.03±0.0
		M6	0.02±0.00	0.02±0.00	0.01±0.00	0.01±0.00	0.01±0.00	0±0.00
		M7	0±0.00	0±0.00	0±0.00	0±0.00	0±0.00	0±0.00
		M8	0±0.00	0±0.00	0±0.00	0±0.00	0±0.00	0±0.00

±: Represent GC-MS analysis errors for isotopomer measurements (n=2)

Isotopomer labeling data for *Pseudomonas aeruginosa* PAO1 WT P (wild type planktonic cells), PAO1 PYEDQ P (biofilm-overexpressing planktonic cells), PAO1 PYHJH P (wild type biofilm-downregulated planktonic cells), PAO1 WT B (wild type biofilm cells), PAO1 PYEDQ B (biofilm-overexpressing biofilm cells), PAO1 PYHJH B (biofilm-downregulated biofilm cells).

			**WT P**	**PYEDQ P**	**PYHJH P**	**WT B**	**PYEDQ B**	**PYHJH B**
alanine	M-57	M0	0.4±0.01	0.39±0.01	0.4±0.01	0.38±0.01	0.39±0.01	0.42±0.01
		M1	0.13±0.01	0.13±0.01	0.12±0.01	0.1±0.01	0.09±0.01	0.08±0.01
		M2	0.47±0.01	0.48±0.01	0.48±0.01	0.51±0.01	0.51±0.01	0.49±0.01
		M3	0.01±0.00	0.01±0.00	0±0.00	0±0.00	0.01±0.00	0.01±0.00
	M-159	M0	0.45±0.01	0.44±0.01	0.45±0.01	0.42±0.01	0.42±0.01	0.45±0.01
		M1	0.52±0.01	0.53±0.01	0.52±0.01	0.55±0.01	0.55±0.01	0.52±0.01
		M2	0.03±0.00	0.03±0.00	0.03±0.00	0.03±0.00	0.03±0.00	0.03±0.00
glycine	M-57	M0	0.93±0.01	0.92±0.01	0.93±0.01	0.9±0.01	0.94±0.01	0.90±0.01
		M1	0.06±0.02	0.06±0.02	0.07±0.02	0.08±0.01	0.05±0.01	0.08±0.01
		M2	0.01±0.00	0.01±0.00	0.01±0.00	0.02±0.00	0.01±0.00	0.02±0.00
	M-85	M0	0.98±0.01	0.97±0.01	0.98±0.01	0.96±0.01	0.98±0.01	0.96±0.01
		M1	0.02±0.01	0.03±0.01	0.02±0.01	0.04±0.01	0.02±0.01	0.04±0.01
valine	M-57	M0	0.17±0.01	0.17±0.01	0.15±0.01	0.13±0.01	0.16±0.01	0.17±0.01
		M1	0.27±0.01	0.27±0.01	0.26±0.01	0.25±0.01	0.26±0.01	0.27±0.01
		M2	0.28±0.01	0.29±0.01	0.28±0.01	0.27±0.01	0.27±0.01	0.27±0.01
		M3	0.27±0.01	0.27±0.01	0.3±0.01	0.34±0.01	0.31±0.01	0.3±0.01
		M4	0.01±0.00	0.01±0.00	0.01±0.00	0.01±0.00	0±0.00	0±0.00
		M5	0±0.00	0±0.00	0±0.00	0±0.00	0±0.00	0±0.00
	M-159	M0	0.19±0.01	0.19±0.01	0.17±0.01	0.15±0.01	0.17±0.01	0.18±0.01
		M1	0.47±0.01	0.47±0.01	0.47±0.02	0.46±0.01	0.47±0.01	0.48±0.01
		M2	0.31±0.01	0.32±0.01	0.33±0.02	0.38±0.01	0.34±0.01	0.32±0.01
		M3	0.02±0.01	0.02±0.01	0.02±0.01	0.02±0.0	0.02±0.0	0.02±0.01
		M4	0±0.00	0±0.00	0±0.00	0±0.00	0±0.00	0±0.00
leucine	M-15	M0	0.07±0.02	0.08±0.02	0.07±0.02	0.05±0.02	0.06±0.02	0.06±0.02
		M1	0.28±0.03	0.28±0.03	0.28±0.03	0.24±0.03	0.26±0.03	0.27±0.03
		M2	0.41±0.03	0.41±0.03	0.4±0.03	0.43±0.03	0.43±0.04	0.4±0.03
		M3	0.21±0.03	0.21±0.03	0.22±0.03	0.25±0.03	0.23±0.03	0.26±0.03
		M4	0.02±0.00	0.02±0.00	0.02±0.00	0.02±0.00	0.01±0.00	0±0.00
		M5	0±0.00	0±0.00	0±0.00	0±0.00	0±0.00	0±0.00
		M6	0±0.00	0±0.00	0±0.00	0±0.00	0±0.00	0±0.00
	M-159	M0	0.16±0.01	0.16±0.01	0.16±0.01	0.13±0.01	0.13±0.01	0.14±0.01
		M1	0.45±0.01	0.45±0.01	0.45±0.01	0.43±0.01	0.44±0.01	0.44±0.02
		M2	0.35±0.01	0.34±0.01	0.35±0.01	0.4±0.01	0.39±0.02	0.38±0.02
		M3	0.04±0.01	0.04±0.0	0.04±0.01	0.04±0.0	0.04±0.01	0.03±0.01
		M4	0.01±0.00	0±0.00	0±0.00	0.01±0.00	0.01±0.00	0±0.00
		M5	0±0.00	0±0.00	0±0.00	0±0.00	0±0.00	0±0.00
isoleucine	M-15	M0	0.1±0.02	0.11±0.02	0.1±0.02	0.08±0.02	0.09±0.02	0.1±0.02
		M1	0.27±0.03	0.26±0.03	0.28±0.03	0.23±0.03	0.24±0.03	0.27±0.03
		M2	0.34±0.03	0.32±0.03	0.32±0.03	0.31±0.03	0.33±0.03	0.31±0.03
		M3	0.23±0.03	0.22±0.03	0.23±0.03	0.27±0.03	0.25±0.03	0.24±0.03
		M4	0.06±0.02	0.08±0.02	0.08±0.02	0.11±0.02	0.09±0.02	0.08±0.02
		M5	0±0.00	0±0.00	0±0.00	0±0.00	0.01±0.00	0±0.00
		M6	0±0.00	0.01±0.00	0±0.00	0±0.00	0±0.00	0±0.00
	M-159	M0	0.14±0.01	0.13±0.01	0.13±0.01	0.1±0.01	0.2±0.01	0.22±0.021
		M1	0.38±0.02	0.38±0.01	0.38±0.01	0.35±0.01	0.27±0.01	0.28±0.01
		M2	0.36±0.02	0.36±0.01	0.36±0.01	0.40±0.01	0.31±0.01	0.30±0.01
		M3	0.12±0.01	0.12±0.01	0.12±0.01	0.15±0.01	0.17±0.01	0.15±0.01

		M4	0±0.0	0±0.0	0±0.0	0±0.0	0.06±0.0	0.05±0.0
		M5	0±0.0	0±0.0	0±0.0	0±0.0	0±0.0	0±0.00
serine	M-57	M0	0.94±0.01	0.94±0.01	0.95±0.01	0.92±0.01	0.94±0.01	0.93±0.01
		M1	0.06±0.01	0.05±0.01	0.04±0.01	0.06±0.01	0.05±0.01	0.05±0.01
		M2	0±0.00	0.01±0.0	0.01±0.0	0.02±0.0	0.02±0.0	0.02±0.0
		M3	0.01±0.00	0±0.00	0±0.00	0±0.00	0±0.00	0±0.00
	M-159	M0	0.97±0.01	0.96±0.01	0.97±0.01	0.96±0.01	0.96±0.01	0.95±0.01
		M1	0.03±0.01	0.04±0.01	0.03±0.01	0.04±0.01	0.04±0.01	0.05±0.01
		M2	0±0.00	0±0.00	0±0.00	0±0.00	0±0.00	0±0.00
threonine	M-57	M0	0.22±0.03	0.23±0.03	0.22±0.03	0.19±0.03	0.2±0.03	0.21±0.03
		M1	0.35±0.03	0.32±0.03	0.35±0.03	0.31±0.03	0.32±0.03	0.34±0.03
		M2	0.29±0.03	0.33±0.03	0.3±0.03	0.34±0.03	0.32±0.03	0.3±0.03
		M3	0.13±0.02	0.13±0.02	0.13±0.02	0.16±0.02	0.16±0.02	0.14±0.02
		M4	0±0.00	0±0.00	0±0.00	0±0.00	0±0.00	0±0.00
	M-159	M0	0.33±0.03	0.28±0.03	0.31±0.03	0.27±0.03	0.28±0.03	0.28±0.03
		M1	0.45±0.03	0.52±0.03	0.48±0.03	0.49±0.03	0.5±0.03	0.5±0.03
		M2	0.21±0.02	0.19±0.02	0.21±0.02	0.22±0.02	0.23±0.02	0.22±0.02
		M3	0±0.00	0.01±0.00	0.01±0.00	0.01±0.00	0±0.00	0±0.00
phenylalanine	M-57	M0	0.81±0.02	0.76±0.02	0.8±0.02	0.73±0.02	0.74±0.02	0.77±0.02
		M1	0.15±0.02	0.17±0.02	0.15±0.02	0.18±0.02	0.17±0.02	0.16±0.02
		M2	0.03±0.01	0.05±0.01	0.04±0.01	0.07±0.01	0.07±0.01	0.05±0.01
		M3	0±0.00	0.01±0.00	0±0.00	0.02±0.00	0.01±0.00	0.01±0.00
		M4	0±0.00	0±0.00	0±0.00	0±0.00	0±0.00	0±0.00
		M5	0±0.00	0±0.00	0±0.00	0±0.00	0±0.00	0±0.00
		M6	0±0.00	0±0.00	0±0.00	0±0.00	0±0.00	0±0.00
		M7	0±0.00	0±0.00	0±0.00	0±0.00	0±0.00	0±0.00
		M8	0±0.00	0±0.00	0±0.00	0±0.00	0±0.00	0±0.00
		M9	0±0.00	0±0.00	0±0.00	0±0.00	0±0.00	0±0.00
	M-159	M0	0.82±0.02	0.78±0.02	0.81±0.02	0.74±0.02	0.76±0.02	0.78±0.02
		M1	0.16±0.02	0.19±0.02	0.17±0.02	0.22±0.02	0.21±0.02	0.19±0.01
		M2	0.02±0.0	0.03±0.0	0.02±0.0	0.04±0.0	0.03±0.0	0.03±0.0
		M3	0±0.00	0±0.00	0±0.00	0±0.00	0±0.00	0±0.00
		M4	0±0.00	0±0.00	0±0.00	0±0.00	0±0.00	0±0.00
		M5	0±0.00	0±0.00	0±0.00	0±0.00	0±0.00	0±0.00
		M6	0±0.00	0±0.00	0±0.00	0±0.00	0±0.00	0±0.00
		M7	0±0.00	0±0.00	0±0.00	0±0.00	0±0.00	0±0.00
		M8	0±0.00	0±0.00	0±0.00	0±0.00	0±0.00	0±0.00
aspartate	M-57	M0	0.23±0.01	0.23±0.01	0.23±0.01	0.2±0.01	0.22±0.01	0.2±0.01
		M1	0.34±0.01	0.33±0.01	0.34±0.01	0.33±0.01	0.33±0.01	0.33±0.01
		M2	0.31±0.01	0.32±0.01	0.3±0.01	0.33±0.01	0.31±0.01	0.31±0.02
		M3	0.12±0.01	0.13±0.01	0.13±0.01	0.14±0.01	0.14±0.01	0.15±0.01
		M4	0±0.00	0±0.00	0±0.00	0±0.00	0±0.00	0±0.00
	M-159	M0	0.31±0.01	0.3±0.01	0.31±0.01	0.28±0.01	0.29±0.01	0.28±0.01
		M1	0.5±0.01	0.49±0.01	0.50±0.02	0.5±0.01	0.5±0.01	0.49±0.01
		M2	0.2±0.01	0.2±0.01	0.20±0.01	0.22±0.01	0.21±0.01	0.22±0.01
		M3	0.01±0.0	0.01±0.0	0±0.00	0±0.00	0±0.00	0±0.00
glutamate	M-57	M0	0.14±0.01	0.14±0.01	0.14±0.01	0.11±0.01	0.12±0.01	0.13±0.01
		M1	0.39±0.01	0.39±0.02	0.38±0.01	0.36±0.01	0.36±0.01	0.38±0.01
		M2	0.36±0.01	0.36±0.01	0.36±0.01	0.38±0.01	0.38±0.01	0.36±0.01
		M3	0.11±0.01	0.11±0.01	0.12±0.01	0.14±0.01	0.14±0.01	0.13±0.01
		M4	0±0.00	0±0.00	0±0.00	0±0.00	0±0.00	0±0.00
		M5	0±0.00	0±0.00	0±0.00	0±0.00	0±0.00	0±0.00
	M-159	M0	0.23±0.01	0.23±0.01	0.22±0.01	0.19±0.01	0.2±0.01	0.22±0.01
		M1	0.48±0.01	0.48±0.01	0.49±0.01	0.48±0.01	0.48±0.01	0.49±0.01

		M2	0.27±0.01	0.27±0.01	0.28±0.01	0.31±0.01	0.31±0.01	0.29±0.01
		M3	0.01±0.00	0.01±0.00	0.01±0.00	0.02±0.00	0.01±0.00	0.01±0.00
		M4	0±0.00	0±0.00	0±0.00	0±0.00	0±0.00	0±0.00
lysine	M-57	M0	0.13±0.02	0.12±0.02	0.11±0.02	0.1±0.02	0.09±0.02	0.09±0.02
		M1	0.26±0.03	0.24±0.03	0.24±0.03	0.23±0.03	0.21±0.03	0.22±0.03
		M2	0.29±0.03	0.31±0.03	0.3±0.03	0.31±0.03	0.29±0.03	0.31±0.03
		M3	0.24±0.03	0.24±0.03	0.25±0.03	0.27±0.03	0.29±0.03	0.27±0.03
		M4	0.09±0.02	0.08±0.02	0.09±0.02	0.11±0.02	0.12±0.02	0.11±0.02
		M5	0±0.00	0.01±0.00	0±0.00	0±0.00	0±0.00	0±0.00
		M6	0±0.00	0±0.00	0±0.00	0±0.00	0±0.00	0±0.00
	M-159	M0	0.15±0.02	0.14±0.02	0.13±0.02	0.12±0.02	0.11±0.02	0.11±0.02
		M1	0.38±0.03	0.4±0.03	0.38±0.03	0.36±0.03	0.35±0.03	0.36±0.03
			0.36±0.03	0.35±0.03	0.36±0.03	0.38±0.03	0.39±0.03	0.38±0.04
			0.11±0.02	0.11±0.02	0.12±0.02	0.14±0.02	0.14±0.02	0.13±0.02
			0.01±0.00	0.01±0.00	0.01±0.00	0.01±0.00	0.01±0.00	0.01±0.00
			0±0.00	0±0.00	0±0.00	0±0.00	0±0.00	0±0.00
histidine	M-57	M0	0.81±0.01	0.84±0.02	0.86±0.02	0.87±0.02	0.87±0.02	0.86±0.01
		M1	0.09±0.02	0.08±0.02	0.08±0.02	0.08±0.02	0.08±0.02	0.08±0.01
		M2	0.08±0.01	0.07±0.01	0.06±0.01	0.05±0.01	0.05±0.01	0.05±0.0
		M3	0.01±0.00	0.01±0.00	0±0.00	0.01±0.00	0±0.00	0.01±0.0
		M4	0±0.00	0±0.00	0±0.00	0±0.00	0±0.00	0±0.00
		M5	0±0.00	0±0.00	0±0.00	0±0.00	0±0.00	0±0.00
		M6	0±0.00	0±0.00	0±0.00	0±0.00	0±0.00	0±0.00
	M-159	M0	0.82±0.02	0.84±0.01	0.85±0.01	0.86±0.02	0.86±0.02	0.85±0.02
		M1	0.07±0.01	0.07±0.01	0.06±0.01	0.07±0.01	0.07±0.02	0.07±0.02
		M2	0.09±0.01	0.07±0.0`	0.06±0.01	0.05±0.00	0.05±0.00	0.06±0.00
		M3	0.01±0.00	0.02±0.00	0.01±0.00	0.01±0.00	0.01±0.00	0.01±0.01
		M4	0±0.00	0±0.00	0±0.00	0±0.00	0±0.00	0±0.00
		M5	0±0.00	0±0.00	0±0.00	0±0.00	0±0.00	0±0.00
tyrosine	M-57	M0	0.81±0.02	0.76±0.01	0.8±0.01	0.74±0.01	0.75±0.01	0.76±0.01
		M1	0.15±0.01	0.18±0.01	0.15±0.01	0.18±0.01	0.17±0.01	0.17±0.01
		M2	0.03±0.01	0.05±0.01	0.04±0.01	0.07±0.01	0.06±0.01	0.05±0.01
		M3	0±0.00	0.01±0.00	0.01±0.00	0.01±0.00	0.01±0.00	0.01±0.00
		M4	0±0.00	0±0.00	0±0.00	0±0.00	0±0.00	0±0.00
		M5	0±0.00	0±0.00	0±0.00	0±0.00	0±0.00	0±0.00
		M6	0±0.00	0±0.00	0±0.00	0±0.00	0±0.00	0±0.00
		M7	0±0.00	0±0.00	0±0.00	0±0.00	0±0.00	0±0.00
		M8	0±0.00	0±0.00	0±0.00	0±0.00	0±0.00	0±0.00
		M9	0±0.00	0±0.00	0±0.00	0±0.00	0±0.00	0±0.00
	M-159	M0	0.81±0.02	0.75±0.02	0.8±0.02	0.72±0.02	0.75±0.01	0.76±0.01
		M1	0.16±0.01	0.20±0.01	0.16±0.01	0.21±0.01	0.2±0.01	0.18±0.01
		M2	0.02±0.01	0.03±0.01	0.03±0.01	0.04±0.01	0.03±0.01	0.03±0.01
		M3	0.01±0.00	0.01±0.00	0±0.00	0.01±0.00	0.01±0.00	0.01±0.00
		M4	0±0.00	0±0.00	0±0.00	0±0.00	0±0.00	0±0.00
		M5	0±0.00	0±0.00	0±0.00	0±0.00	0±0.00	0±0.00
		M6	0±0.00	0±0.00	0±0.00	0±0.00	0±0.00	0±0.00
		M7	0±0.00	0±0.00	0±0.00	0±0.00	0±0.00	0±0.00
		M8	0±0.00	0±0.00	0±0.00	0±0.00	0±0.00	0±0.00

±: Represent GC-MS analysis errors for isotopomer measurements (n=2)

Table S2. List of flux values in the central metabolism of *Pseudomonas Aeruginosa* biofilm and planktonic estimated from the 13C MFA model.

Reactions
Glucose == G6P
G6P == F6P
F6P == FBP
FBP == DHAP + GAP
DHAP == GAP
GAP == 3PG
3PG == PEP
PEP == PYR
PYR == AceCoA + CO_2
Acetate == AceCoA
AceCoA + OAA == CIT
CIT == ICIT
ICIT == AKG + CO_2
AKG == SUC + CO_2
SUC == SucCoA
SUC == FUM
FUM == MAL
MAL == OAA
MAL == PYR + CO_2
PEP + CO_2 == OAA
G6P == 6PG
6PG == Ru5P + CO_2
Ru5P == R5P
X5P == Ru5P
GAP + S7P == X5P + R5P
E4P + F6P == GAP + S7P
GAP + F6P == X5P + E4P
6PG == GAP + PYR
ICIT == GLX + SUC
GLX + AceCoA == MAL
0.447*ALA+0.257*ARG+0.210*ASN+0.210*ASP+0.080*CYS+0.229*GLU+0.229*GLN+0.533*GLY+0.082*HIS+0.253*ILE+0.392*LEU+0.298*LYS+0.134*MET+0.161*PHE+0.192*PRO+0.188*SER+0.221*THR+0.049*TRP+0.120*TYR+0.368*VAL+0.141*G6P+0.754*R5P+0.129*GAP+0.619*G3P+0.051*PEP+0.083*PYR+2.510*AceCoA+0.087*AKG+0.340*OAA+0.443*Methylene_THF+33.247*ATP+5.363*NADPH==39.68*Biomass+1.455*NADH
0.3*(0.447*ALA+0.257*ARG+0.210*ASN+0.210*ASP+0.080*CYS+0.229*GLU+0.229*GLN+0.533*GLY+0.082*HIS+0.253*ILE+0.392*LEU+0.298*LYS+0.134*MET+0.161*PHE+0.192*PRO+0.188*SER+0.221*THR+0.049*TRP+0.120*TYR+0.368*VAL)+0.27*S7P+1.93*G6P==EPS
